# Will executive equity incentives affect investor relations management: Empirical evidence from Chinese listed companies

**DOI:** 10.3389/fpsyg.2022.968677

**Published:** 2022-12-19

**Authors:** Xiaofei Shi, Zunhu Liu, Yuanfang Wang

**Affiliations:** ^1^School of Business Administration, Hebei University of Economics and Business, Shijiazhuang, China; ^2^School of Accounting, Capital University of Economic and Business, Beijing, China

**Keywords:** executive equity incentive plans, investor relations management, telephone communication, network communication, on-site communication

## Abstract

How to improve the level of investor relationship management of listed companies and establish trust relationship with investors is an important research issue for enterprises in the capital market. From the perspective of optimal contracting theory, we construct a theoretical model to assess how executive equity incentive plans (EEIPs) affect enterprises’ investor relationship management. For the analysis purpose, this study looks into panel data issues in depth by using approaches the fixed effect (FE) method, and the study employs the propensity score matching (PSM), instrumental variable method, and core indicator substitution method to test the robustness of the conclusions. Based on the panel data of Chinese A-share listed companies from 2014 to 2019, our baseline results indicate that EEIPs improves investor relations. This positive effect mainly exists in stock options, rather than restricted stocks. In the sample of enterprises implementing EEIPs, the intensity of executive equity incentive is positively correlated with investor relationship management. Further research shows that EEIPs mainly through telephone communication, network communication and on-site communication to achieve the impact of listed companies investor relationship management. These findings enriches the economics of executive equity incentives from the perspective of investor relations management. At the same time, it has certain guiding significance for improving the design of the incentive system for corporate executives and improving the information efficiency of the capital market.

## Introduction

How to design an effective management compensation contract and motivate the management to serve the interests of shareholders has always been an important issue in corporate governance. Due to differences in equity structure and market development, early equity incentives were only used in corporate compensation systems in Western countries. For Chinese enterprises, this compensation incentive method is still in the exploratory stage (Lv and Zhang, 2011; Zong et al., 2013). On December 31, 2005, the China Securities Regulatory Commission issued the “Administrative Measures for Equity Incentives of Listed Companies (Trial),” which clarified the status and scope of application of equity incentives, and gave some regulations and guidelines for the establishment of equity incentive plans. In this context, many listed companies have tried to implement equity incentive plans. The “Statistical and Analysis Report on Equity Incentive Practice of A-Share Listed Companies in 2021” shows that in 2021, 808 A-share listed companies announced a total of 826 equity incentive plans, an increase of 82.74% compared with 452 cases in 2020. The confirmation of the gradual normalization of incentives. With the development of the securities market and the improvement of the equity incentive system, the equity incentive plans of listed companies have become more widely used, which will inevitably have a certain impact and impact on the traditional employee compensation incentive model and corporate governance system.

Whether the information efficiency of capital market can be improved is an important part of the research on the economic consequences of executive equity incentives (Solomon and Soltes, 2015; Xiao et al., 2017). Under the perfect capital market hypothesis, there is no transaction information cost and agency problem in the market, and there is an absolute information symmetry between investors and companies. However, the capital market is not ideal, it has transaction and agency costs. The agency theory stated that the agent has more information than the principal, and this information asymmetry will adversely affect the effectiveness of the agent’s service for the principal’s interests. Investors (especially small and medium-sized shareholders) need help from company executives or securities analysts to provide more capital information for value evaluation and make investment decisions. Information inefficiency will affect investors’ value judgments and form expected deviations in company valuations, thereby affecting stock trading (Li and Wang, 2008). On the one hand, based on signaling theory, executive equity incentive plans(EEIPs) send signals to the outside world that executives are making greater efforts, which may change the value of the company (Himmelberg et al., 2000; Sheng et al., 2016), it will attract investors’ attention; On the other hand, under the hypothesis of optimal contracting theory, EEIPs links the wealth of executives with the performance of the company’s stock market, which largely depends on the amount of information held by investors, so the company’s executives are motivated to release more information to investors psychologically (Nagar and Nanda, 2003; Zhao, 2019). Information disclosure is an important content of investor relations management, in contrast to the traditional one-way communication, investor relations management (IRM) focused on building the bidirectional information channels of communication with investors, through the establishment of an investor relationship network platform, telephone communication, holding performance briefings, and investor research reception, etc., we can achieve multi-dimensional and proactive relationships with investors and enhance investor recognition (Li et al., 2006; Ma et al., 2014). At the same time, a high level of investor relations management will help listed companies accumulate reputation capital, reduce equity financing constraints, and improve corporate value (Li et al., 2007; Ma et al., 2015; Quan et al., 2017).

**Figure 1:**
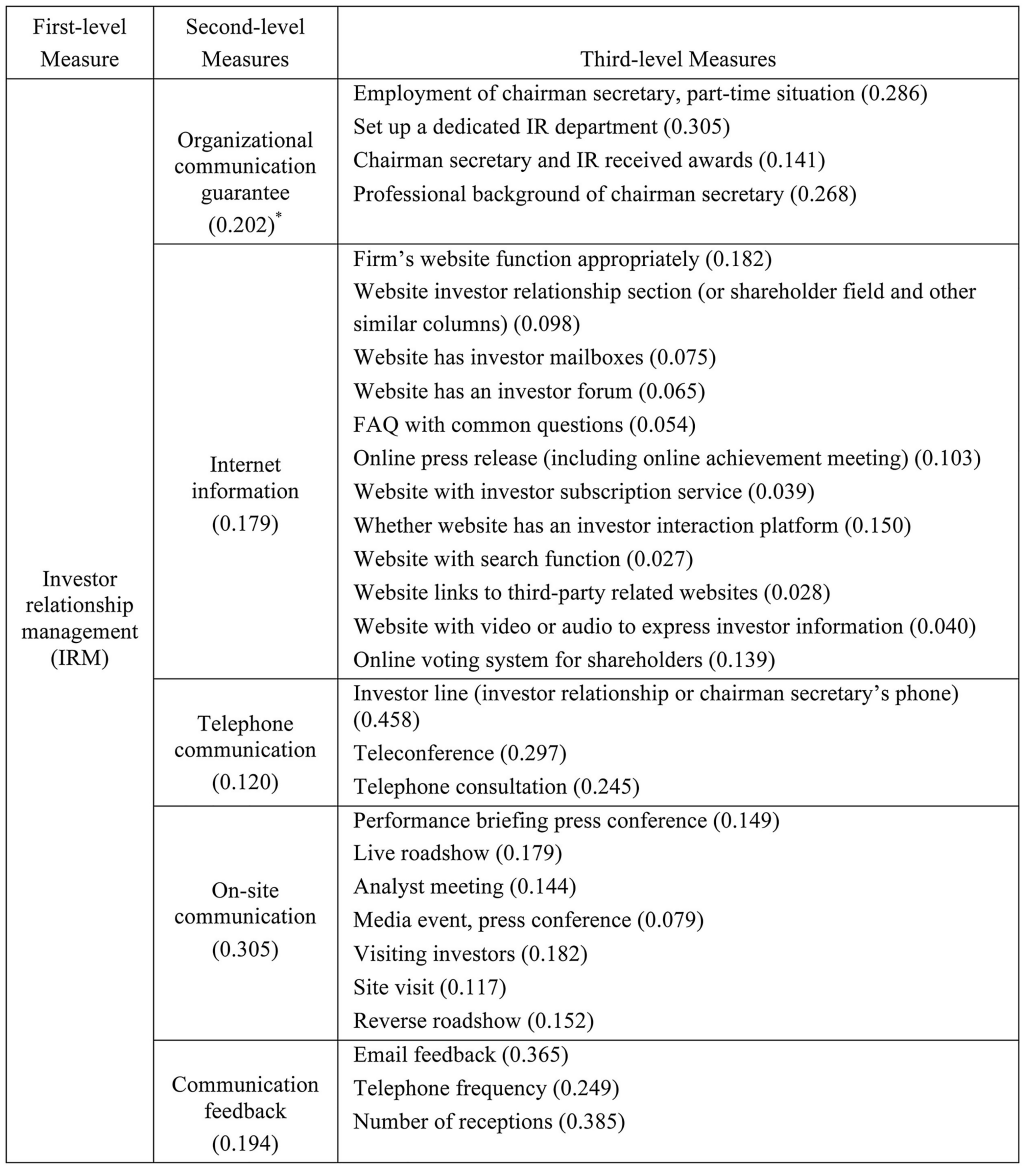
Investor relationship management index measures.

The objective of this paper is to examine whether executive equity incentive plans motivate a firm’s investor relations management. EEIPs as an important way of executive compensation incentive, plays a positive role in the company’s performance disclosure and investor communication (Ma and Zhang, 2020). However, some scholars pointed out that the equity incentive scheme is difficult to effectively motivate executives due to its welfare effect, may cause executives to pay too much attention to short-term stock price fluctuations and the company’s short-term performance, and to ignore investment in investor relations (Hong and Huang, 2003). Therefore, for the new ecology of China’s capital market under the background of registration reform, whether equity incentive can promote investor relationship management is an empirical issue. At present, the academic circle has not given enough attention and demonstration. Furthermore, based on Ma et al. (2014) indicator classification of IRM (As shown in Figure 1), this paper analyzes the impact of executive equity incentive plans on specific dimensions of investor relationship management. We hope to supplement the existing equity incentive research gaps in the field of investor relations.

Using the data of IRM and EEIPs among Chinese firms from 2014 to 2019, we document that firms with EEIPs have greater investor relationship compared to those without EEIPs and this positive effect mainly exists in stock options, rather than restricted stocks. In the sample of enterprises implementing EEIPs, the intensity of executive equity incentive is positively correlated with investor relationship management. For the analysis purpose, this paper employs fixed-effect (FE) model was used in this paper. In addition, For more robust results, this paper employs the propensity score matching (PSM), instrumental variable method, and core indicator substitution method to test the robustness of the conclusions. Further research shows that EEIPs mainly achieves its influence on investor relationship management through telephone communication, network communication and on-site communication, among which enterprises are more inclined to adopt on-site communication to interact with investors.

Our findings make two contributions. First, from the perspective of executive equity incentive, it solves the problem of information disclosure and two-way communication between investors. Most of the existing literature focuses on corporate performance (Hanlon et al., 2003; Zhen et al., 2021), market performance (Kato et al., 2004; Lv et al., 2009), investment behavior (Lu et al., 2015), risk appetite (Low, 2008; Armstrong and Vashishtha, 2012; Li and Zhang, 2014) and earnings Management (Cheng and Warfield, 2005; Liu et al., 2017) studied the economic consequences of equity incentive, and few literature related to the relationship between EEIPs and two-way information communication of investors. Based on the perspective of investor relationship management, this paper studies the information interaction behavior and interaction path between EEIPs and external investors, hoping to provide some help for the management to choose the information disclosure mode and improve the information efficiency of the capital market. Second, it adds new evidence for the exploration of influencing factors of investor relationship management. The importance of investor relationship management has been recognized by both theoretical and practical circles, but there are few researches on how to improve the level of investor relationship management. Research on investor relations in China started late, and many key areas have not been carefully analyzed (Li, 2013). At present, scholars’ research on investor relationship management focuses on four aspects: corporate financial operation status, corporate governance level, corporate governance environment and financing motivation. We attempts to make an in-depth analysis of the influence of investor relationship management level from the perspective of executive equity incentive, in order to effectively expand the research on IRM.

## Literature review and research hypothesis

### Executive equity incentive plans and investor relationship management

The core idea of management incentive is agency theory. The separation of ownership and management in modern enterprises requires shareholders to entrust management to maximize the value of the company, that is, maximize the wealth of shareholders. However, due to the self-interest of people, agents will act in accordance with their own interests, thereby damaging the interests of shareholders, that is, agency costs. The source of agency cost is information asymmetry, and the behavior of management cannot be fully supervised by shareholders. This kind of agency problem is also called moral hazard. The main goal of establishing the executive compensation incentive plan is to solve the moral hazard and link the interests of the management with the interests of investors. According to the optimal contract theory, the board of directors can encourage management to maximize shareholder wealth by designing a reasonable compensation contract. Therefore, an effective compensation contract can alleviate the agency conflict between shareholders and management, make shareholders’ interests consistent with managers’ interests, reduce agency costs, and improve company value. The incentive intensity of an enterprise can reflect the efforts and productivity of managers. The higher the salary paid to executives, the stronger the sensitivity of salary performance, and the more incentive managers can engage in activities conducive to the value of the company. Investor relationship management is an important factor to enhance enterprise value. In addition, From the perspective of the theory of human capital property rights, the way to realize equity incentive is to make the business operators have certain property rights through equity, so as to closely combine the interests of shareholders, the interests of the company and the personal interests of the operators, and make the business operators pay more attention to the investor relationship management of the company from the perspective of obtaining sustainable benefits.

At present, scholars’ research on the influence of executive equity incentive on investors mainly focuses on information disclosure. The existing research views are mainly divided into two categories. First, under the hypothesis of optimal contracting theory, EEIPs combine the interests of corporate executives with market performance, which can effectively alleviate the conflicts of interest between shareholders and management (Jensen and Meckling, 1976). At the same time, the “synergy of interests” effect generated by incentives will improve the tendency of executives to disclose private information, so as to reduce the noise component in stock prices unrelated to their own efforts (Muslu et al., 2015; Kim et al., 2019). Second, under the hypothesis of managerial power theory, equity incentive is not a governance mechanism to alleviate agency problems, but will become a tool of “management rent-seeking” (Bebchuk and Fried, 2003; Lin and Ailsa, 2010). EEIPs makes executive compensation closely related to the performance of the company’s stock price, which may lead to management information manipulation while motivating the management to work hard, thus reducing the ability of investors to predict the prospect of the enterprise (Lundholm and Myers, 2002; Choi et al., 2011; Muslu et al., 2015).

Will management disclose more private information to investors after listed company companies implement EEIPs? Nagar and Nanda (2003) to the frequency of the management performance forecast as the proxy variable, the number of information disclosure by analysts to management in information disclosure of rating as the quality of information disclosure to study the effect of equity incentive for information disclosure, and found that after the implementation of EEIPs, the frequency of release management earnings forecast more, and the company information disclosure quality is higher. At the same time, Almazan and Motta (2008) also pointed out that, driven by the motivation of increasing the stock value to obtain more benefits, the equity-motivated management was willing to release more information to attract the attention of the investment market.

The core of investor relationship management lies in information disclosure and two-way interactive communication for investors (Ma et al., 2015). In the above discussion, we has pointed out that EEIPs can promote the information disclosure of investors, but there are few literature studies on whether the executives who are motivated have more interaction and communication with outside investors. Almazan and Motta (2008) found that EEIPs made the management willing to provide more informal communication for the market. When the management expected investors to track and dig the private information of the company, the information interaction between the management and investors would be more frequent. Xiao and Yang (2018) point out that EEIPs is conducive to reducing the space of executive “agent opportunism.” However, in enterprises with less opportunistic management behaviors, listed companies have more information disclosure and two-way communication to the outside world [38]. In what way does equity incentive promote the interaction between executives and investors? When Zhao (2019) studied the arbitrage space of equity incentive on management private information, he found that after the implementation of EEIPs, listed companies would conduct more investor investigations with larger scale and lower information asymmetry. In addition, analysts, as an important medium connecting listed companies and external investors, companies implementing executive equity incentive plans will attract more analysts to follow and improve the information flow efficiency of the capital market (Wang et al., 2019).

As an important mechanism of corporate governance, investor relations management main function is to reduce the information asymmetry between listed companies and the investment community, through to the investors, analysts and other disclosure the company’s performance and related information, promote capital market for the company’s understanding and recognition, ultimately maximize the value of the company (Yang et al., 2007; Song and Thakor, 2008; Quan et al., 2016). Management performance forecast, investor survey reception and information flow between analysts and enterprises are all important contents of investor relationship management (Li et al., 2006; Kirk and Vincent, 2014; Ma et al., 2014).

However, EEIPs may provide an incentive for managers to pay too much attention to short-term stock price fluctuations, which will result in managerial myopia and accounting manipulative activities (Cheng and Warfield, 2005; Armstrong and Vashishtha, 2012). And an increase in the level of EEIPs may further increase managers’ rights, which consequently produces managerial private rent-seeking and a serious agency problem (Shi et al., 2017). Higher managerial equity incentives imply that managers must bear greater risk costs, and this reduces their willingness to disclose information and exacerbated information asymmetry. Therefore, EEIPs may damage investor relationship management.

Although equity incentive may have both positive and negative effects on investor relationship management, we still predict that equity incentive has positive effect in China. According to optimal contracting theory, the establishment of a synergistic mechanism for the interests of executives, enterprises and shareholders will intrinsically drive executives to increase the frequency of information exchange between enterprises and investors (Muslu et al., 2015; Kim et al., 2019). In the process of increasing incentive intensity, the synergy consciousness of executives is reflected in increasing focus on enhancing corporate value (Kato et al., 2004), investor relationship management plays an important role in the embodiment of corporate value. we presume that EEIPs can promote the level of investor relationship management of listed companies, which is embodied in the following three aspects: First, interest driven. In order to improve the market value expectation of the capital market and obtain excess stock returns, executives will be more willing to disclose private information to investors through different media (Almazan and Motta, 2008). Second, interest substitution. Using data of Chinese firms, Feng and Zhao (2012) and Liang (2016) believe that there is a substitution effect between EEIPs and in-service consumption. When the incentive intensity is low, executives will focus more on obtaining short-term benefits. Hong and Huang (2003) hold the same view on the interest substitution behavior in equity incentive, and put forward that when internal managers hold fewer shares, they will lose the potential benefits of informed transaction if they do investor relations well, so they seldom pay attention to investor relations policies. With a high incentive level, executives are willing to improve investor relations to improve stock liquidity and asset liquidity. Third, role change. According to Stulz (1988), EEIPs makes the executives complete the transformation from employees to shareholders, and it is easy to realize the consistency of the long-term objective function between executives, enterprises and shareholders. In this process, manager opportunism is further compressed, which will promote the level of investor relationship management (Xiao et al., 2007). Consistent with these arguments, Ma and Zhang (2020) find that EEIPs can promote investor relation in China. Consequently, the following research assumptions are proposed:

*Hypothesis* 1. Compared with listed companies that have not implemented EEIPs, the level of investor relationship management of companies that implemented EEIPs is higher.

### Equity incentive models and investor relationship management

Stock option and restricted stocks are the two main equity incentive models of Chinese listed companies after non-tradable shares reform (Li, 2008). Xu and Xu (2012) studied the equity incentive plans of Chinese listed companies from 2006 to 2009, and found that state-owned enterprises, oriented by profit space, preferred restricted stocks, while high-growth companies preferred stock options. The risk taking mechanism, the symmetry of rights and obligations, and the symmetry of incentive and punishment are the essential differences between the two kinds of equity incentive modes (Bryan et al., 2000). These basic differences determine the different incentive objects applicable to them. Domestic and foreign research results show that in the compensation incentive system, the effect of granting stock options to executives is better than restricted stock (Dodonova and Khoroshilov, 2006; Liu and Ma, 2013; Lim, 2014; Xu and Chen, 2019). Under the incentive model of restricted stock, managers must meet the requirements of holding years and performance assessment before they can realize the earnings. In other words, restricted stock makes executives focus on achieving the established performance requirements. However, under stock option incentive mode, managers do not have such concerns when choosing investment projects. Even if the stock price falls, managers can choose not to take the right to avoid their own losses, which is more conducive to encouraging the object to take risks (Sanders and Hambriek, 2007) and weakening the opportunism tendency of management (Low, 2008), thus promoting the improvement of investor relationship management level of listed companies. Consequently, we propose the following research assumptions:

*Hypothesis* 2. Compared with listed companies that implement restricted stock incentive, the level of investor relationship management of companies that implement stock option incentive is higher.

## Materials and methods

### Sample selection

We studies the influence of executive equity incentive system on investor relationship management of listed companies in China. In this paper, Chinese a-share listed companies from 2014 to 2019 are selected as samples. According to the research needs, the initial samples are processed as follows: (1) the financial industry is excluded, as well as the samples of companies that are ST, *ST and PT during the sample period; (2) Eliminate the company samples with missing data of major variables; (3) Outlier processing was performed after the data distribution morphology analysis of the main variables; (4) The samples of companies whose equity incentive mode is stock appreciation right and compound mode are excluded. Finally, a total of 16,593 observation values were obtained from 3,355 listed companies, including 1,256 observation values of executive equity incentive and 15,337 observation values of non-executive equity incentive. The equity incentive data, corporate financial data and other information used in this paper come from CSMAR database and RESSET Financial Research database, and the investor relationship management measurement index comes from IRIINK database of China Academy of Corporate Governance.

### Research methodology

In order to test our hypotheses, Hausman Test was used to judge panel data using random effect model or fixed effect model. The test results showed that the *p* value was 0.0000, so the original hypothesis was strongly rejected and fixed effect model should be used instead of random effect model. Therefore, we set the following model.

Model 1.


$$\begin{array}{l} {\text{IRII}}_{i,t}=\alpha_{0}+\alpha_{1}\;{\text{EIIPs}}_{i,t-1}+\alpha_{2}{\text{Controls}}_{i,t-1}\\ \;+\;£\text{ Year}+£\text{ Industry}+\varepsilon \end{array}$$

Where i indexes the firm and t indexes time. IRIIi, t represents the level of investor relations management of listed companies. We divide EEIPs into three dimensions: EI, EI_I and EI_M. EI is a dummy variable that is equal to one if the firm is implementing executive equity incentive plans in a given year, and zero otherwise. EI_I is executive equity incentive intensity, EI_M is executive equity incentive model, the stock option mode is 1 and the restricted stock mode is 0. Controls *i*, *t*-1 represents all control variables, Year, Industry represent year and industry fixed effects, respectively. A detailed definition for each variable used in this paper is provided in Table 1.

**Table 1:** Variable definitions.

Variable symbol	Variable name	Variable description
IRII	Investor Relations Management Index	Investor relationship management measure: Drawn from the investor relationship management index of listed Chinese companies from the Investor Relations Management Laboratory of Nankai University Corporate Governance Center
EI	Dummy Variable of Equity Incentive	The equity incentive implemented during the year is 1, otherwise it is 0
EI_I	Equity Incentive Intensity	Model (2) calculation
EI_M	Equity Incentive Model	The stock option mode is 1 and the restricted stock mode is 0
Size	Firm Size	Natural logarithm of total assets of the company during the year
Equity	Executive Shareholding Level	Natural logarithm of the number of shares held by directors, supervisors and senior management
Lev	Financial Leverage	Total liabilities/Total assets
ROA	Return on Assets	Net profit/total assets balance
Growth	The Growth Rate of Main Business	(The main business income this year-The main business income of last year)/The main business income at the beginning of this year
Top1	The Largest Shareholder’s Shareholding Ratio	Number of shares held by the largest shareholder/total number of company shares
Cr_10	Shareholding Ratio of the Top Ten Shareholders	Number of shares held by the top ten shareholders/total number of shares of the company
Soe	Nature of Property Rights	State-owned:1, private: 0
Ins	Institutional Investor Shareholding Ratio	Number of shares held by each institution/total number of company shares
Inde	Board Independence	Number of independent directors/total number of board of directors
Year	Year	Control dummy variable
Ind	Industry	Control dummy variable

### Dependent variable

In our models, the dependent variable is investor relations management (IRM). The National Investor Relations Association of the United States (NIRI) defines investor relations management as a strategic management behavior that uses financial, communication, marketing and other means, complies with securities laws and regulations, and is committed to forming the most effective two-way communication among companies, financial institutions and other investors, so as to realize the fair valuation of the company’s shares. This definition emphasizes the two-way interaction between listed companies and capital market participants. In the early days, the measurement of investor relations often subdivided interaction and communication into one aspect, such as the construction of investor relations websites (Bollen et al., 2006), performance news release (Meijer and Kleinnijenhuis, 2006), and the interaction between analysts and listed companies (Rowbottom et al., 2004). Although it can focus on reflecting a certain problem, it lacks a panoramic summary. Based on the special national conditions of China’s listed companies and comprehensiveness of investor relations indicators, we choose IRIINK index as the measurement index of investor relationship management. The measure of investor relationship management comes from the investor relationship management index (IRII^NK^) of Listed Companies in the Investor Relations Management Laboratory of Nankai University Corporate Governance Center. We use IRII^NK^ to analyze the status of interaction and communication between listed companies and investors, as shown in Figure 1. According to the “Guidelines for Investor Relations of Listed Companies” issued by the China Securities Regulatory Commission, the evaluation system of IRII^NK^ is set to include 5 secondary indicators and 29 tertiary indicators. First，the importance of the indicators at all levels of the Investor Relations Interaction Index was scored by 15 experts according to the Delphi Method, next, the opinions were summarized and revised repeatedly to reduce the influence of bias in individual subjective judgments, then, the project team used the Analytic Hierarchy Process (AHP) quantifies the subjective judgment, and determines the weight of each index, last, the IRII^NK^ of each sample company is determined by manually collecting and calculating relevant data from the annual report and the company’s website. The detailed calculation method of the indicators refers to Ma et al. (2014).

### Explanatory variables

The explanatory variables is executive equity incentive plans. Referring to the studies of Sanders and Hambriek (2007) and Wu et al. (2022), we adopts EI, EI_I and EI_M to measure EEIPs. EI_I is executive equity incentive intensity, according to the method of Bergstresser and Philippon (2004), we uses the ratio of the increment in the value of stock and stock options held by executives to their total compensation when the stock price rises 1% to measure the intensity of equity incentive. The calculation formula is as follows:


$$EI\_I_{i,t}=\frac{0.01\times \text{Price}\;i,t\times \text{Shares}\;i,t+\text{Options}\;i,t}{0.01\times \text{Price}\;i,t\times \text{Shares}\;i,t+\text{Options}\;i,t+\text{Cashpay}\;i,t}$$

Where *i* indexes the firm and *t* indexes time. Price *i*, *t* is the closing price of company i’s stock at the end of *t*, Shares *i*, t and Options *i*, *t* are the number of stocks and options held by company executives (including directors and supervisors) in year *t*, and Cashpay *i*, *t* is executives the cash salary of the year, including annual salary and various allowances.

### Control variables

We select factors that may have an impact on investor relationship management as control variables. According to the existing literature, debt level (Zhao, 2009), firm size (Kirk and Vincent, 2014), proportion of independent directors (Zhang and Chen, 2009), shareholding concentration (Zhao, 2011), shareholding by institutional investors (Xiao et al., 2007) and other factors will affect investor relations management. Among them, ownership concentration is negatively correlated with investor relationship management, while other factors are positively correlated with investor relationship management. Listed companies are more inclined to actively communicate with investors when they have good financial operating conditions and perfect corporate governance mechanism (Ma and Zhang, 2020). So we select firm size, executive shareholding level, financial leverage, return on assets (ROA), the growth rate of main business, the largest shareholder’s shareholding ratio (Top1), shareholding ratio of the top ten shareholders (Cr_10), nature of property rights, institutional investor shareholding ratio as control variables. Considering the potential time-specific and any unobservable industry characteristics shocks to a firm’s investor relationship management activities, we also include year and industry fixed effects. All variable definitions are shown in Table 1.

## Results

### Major results

Tables 3 and 4 display the descriptive statistics of our sample. Table 2 shows the basic descriptive statistical results of the main variables, including sample observations, averages, and standard deviations. It can be seen that among all the observations, the sample of executive equity incentive companies accounted for about 7.6% of the total sample, with a standard deviation of 0.265. Among them, the average intensity of executive equity incentives was 0.549 and the standard deviation was 0.366. It can be seen that the implementation of executive equity incentive plans (EEIPs) by listed companies in China is still in the exploratory stage, and the degree of incentives between different companies is quite different. Companies using stock options as the incentive model account for approximately 25.6% of all incentive samples, indicating that the EEIPs of listed companies in China are dominated by restricted stock models. The IRII index has an average of 0.303 and a standard deviation of 0.128, indicating that the average level of investor relationship management of listed companies in China is relatively low and there are obvious differences in the level of investor relationship management between companies. In addition, Table 2 also reports the test results (columns (4) and (5)) of the mean difference between the sample of non-executive equity incentive companies and the sample of executive equity incentive companies. The results of the mean difference test showed that the sample investor relationship management index without EEIPs was lower than the average, and the enterprises implementing EEIPs were significantly higher than the un-incentive enterprises. Preliminary analysis shows that executive equity incentive plans may have a positive role in promoting the level of corporate investor relationship management. Among other control variables, the average asset-liability ratio of the sample companies is approximately 41.9%, the average shareholding ratio of the largest shareholder is approximately 34.3%, the average shareholding ratio of the top ten shareholders is approximately 5.93%, and the return on assets is approximately 3.7%. The average growth rate of main business income is about 27.3%, the average shareholding ratio of institutional investors is about 43.2%, the average ratio of independent directors on the board of directors is about 37.7%, and the average number of professional committees is about 3.952. The test of the mean difference of the control variables is basically also more significant, which shows that the selection of the control variables in this paper is reasonable.

**Table 2:** Descriptive Statistics.

Variable	All samples			EI = 0	EI = 1
				*N* = 15,337	*N* = 1,256
	*N*	Mean	Std. Dev.	Mean	Mean
	(1)	(2)	(3)	(1)	(2)
IRII	16,593	0.303	0.128	0.3	0.326^***^
EI	16,593	0.076	0.265		
EI_I	1,256	0.549	0.366		
EI_M	1,256	0.256	0.437		
Size	16,593	9.651	0.584	9.656	9.583^***^
Equity	16,593	5.552	3.151	5.421	7.156^***^
Lev	16,593	0.419	0.205	0.421	0.393^***^
ROA	16,593	0.037	0.123	0.035	0.055^***^
Growth	16,593	0.273	2.112	0.268	0.322
top1	16,593	0.343	0.148	0.344	0.325^***^
Cr_10	16,593	0.593	0.152	0.591	0.607^**^
Soe	16,593	0.343	0.475	0.36	0.143^***^
Ins	16,593	0.432	0.248	0.436	0.377^***^
Inde	16,593	0.377	0.055	0.376	0.383^**^

^***^, ^**^, ^*^represent the significance level of 1, 5, 10%, respectively.

**Table 3:** Correlation matrix (equity incentive, EI).

	IRII	EI	Size	Equity	Lev	ROA	Growth	Top1	Cr_10	Soe	Ins	Inde
IRII	1											
EI	0.052^***^	1										
Size	0.052^***^	0.033^***^	1									
Equity	0.227^***^	0.146^***^	0.164^***^	1								
Lev	0.059^***^	0.035^***^	0.519^***^	−0.215^***^	1							
ROA	0.033^***^	0.043^***^	−0.015^*^	0.057^***^	0.182^***^	1						
Growth	0.016^**^	0.007	0.036^***^	−0.014^*^	0.044^***^	0.023^***^	1					
Top1	0.094^***^	0.033^***^	0.205^***^	−0.258^***^	0.052^***^	0.087^***^	0.003	1				
Cr_10	0.038^***^	0.027^***^	0.117^***^	−0.005	0.092^***^	0.141^***^	0.031^***^	0.612^***^	1			
Soe	0.140^***^	0.121^***^	0.375^***^	−0.543^***^	0.288^***^	0.036^***^	0.008	0.229^***^	0.039^***^	1		
Ins	0.100^***^	0.063^***^	0.456^***^	−0.504^***^	0.224^***^	0.041^***^	0.029^***^	0.497^***^	0.452^***^	0.432^***^	1	
Inde	0.019^**^	0.028^***^	0.001	0.018^**^	0.004	−0.01	0.01	0.044^***^	0.030^***^	0.051^***^	0.061^***^	1

^***^, ^**^, ^*^ represent the significance level of 1, 5, 10%, respectively.

**Table 4:** Correlation matrix (EI_I and EI_M).

	IRII	EI_I	EI_M	Size	Equity	Lev	ROA	Growth	Top1	Cr_10	Soe	Ins	Inde
IRII	1												
EI_I	0.153^***^	1											
EI_M	0.054^*^	0.131^***^	1										
Size	0.0290	0.420^***^	0.202^***^	1									
Equity	0.154^***^	0.716^***^	0.166^***^	0.231^***^	1								
Lev	−0.057^**^	0.281^***^	0.155^***^	0.585^***^	0.162^***^	1							
ROA	0.0100	0.116^***^	0.158^***^	0.082^***^	0.080^***^	0.326^***^	1						
Growth	0.0310	0.0240	−0.0310	0.076^***^	0.0220	0.073^***^	0.0160	1					
Top1	0.095^***^	0.203^***^	0.0460	0.133^***^	0.242^***^	0.102^***^	0.063^**^	0.003	1				
Cr_10	0.132^***^	0.063^**^	−0.0190	−0.0200	0.079^***^	−0.0450	0.157^***^	0.0270	0.591^***^	1			
Soe	0.087^***^	0.487^***^	0.136^***^	0.411^***^	0.515^***^	0.248^***^	−0.049^*^	0.0390	0.204^***^	0.0190	1		
Ins	0.074^***^	0.648^***^	0.117^***^	0.453^***^	0.515^***^	0.245^***^	0.0430	0.0370	0.400^***^	0.351^***^	0.382^***^	1	
Inde	0.0310	0.006	0.052^*^	−0.03	−0.01	−0.054^*^	0.066^**^	−0.030	0.068^**^	−0.0130	0.008	0.053^*^	1

^***^, ^**^, ^*^represent the significance level of 1, 5, 10%, respectively.

In this paper, Pearson test was used to analyze the correlation of executive equity incentive, executive equity incentive intensity, executive equity incentive model, investor relationship management and other related variables. The results show that the correlation coefficient of EI and IRII is 0.052, and it is significant at the level of 1%, indicating that companies implementing EEIPs have the motivation to improve the level of investor relations management. Table 4 reports the correlation coefficients between the main variables of the sample implementing EEIPs. The results show that the correlation coefficient of executive equity incentive intensity (EI_I) and IRII is 0.153, and it is significant at the level of 1%, which to some extent indicates that as the intensity of executive equity incentives increases, the company’s investor relations The management level may continue to improve, which supports the Hypothesis 1. The correlation coefficient between the incentive model (EI_M) and IRII is 0.054, and it is significant at the level of 10%, indicating that from the perspective of incentive methods, stock options may have a better incentive effect on the improvement of investor relations management, which supports the Hypothesis 2.

Based on whether there are executive equity incentive plans in enterprises, enterprises are divided into executive equity incentive enterprises and non-executive equity incentive enterprises. In this part, this paper tests whether EEIPs has a higher level of investor relationship management to verify Hypothesis 1.

Table 5 lists the multiple regression results of EEIPs and investor relationship management. In column (1), the regression coefficient of IRII and EI is 0.019, and it is significant at the level of 1% (*β* = 0.019, *p* < 0.01), which is in line with optimal contracting theory expectation, indicating that under the implementation of equity incentive, listed companies have a trend to improve the level of investor relations management. The regression results show that under the same other conditions, companies that implementing equity incentive have a higher level of investor relations management after implementation than companies that have not implemented equity incentive, which supports Hypothesis 1. This result shows that the implementation of EEIPs of listed companies will establish a “profit synergy” mechanism, compress the management opportunism space, and internally drive executives to enhance the information interaction between the company and external investors. For investors, the release of equity incentive plans for executives by listed companies is also a signal of a reduction in information asymmetry. At the same time, this kind of salary incentive system is beneficial to the company’s market value management to a certain extent and raises the expectations of the capital market.

**Table 5:** Regression results of executive equity incentive plans (EEIPs) and investor relations management.

	(1)	(2)	(3)
	IRII	IRII	IRII
EI	0.0119^***^		
	(3.45)		
EI_I		0.0525^***^	
		(2.77)	
EI_M			0.0236^***^
			(2.69)
Size	0.0511^***^	0.0449^***^	0.0394^***^
	(23.13)	(4.47)	(3.89)
Equity	0.0065^***^	0.0039	0.0089^***^
	(16.35)	(1.34)	(3.60)
Lev	−0.0521^***^	−0.098^***^	−0.0969^***^
	(−9.07)	(−3.58)	(−3.54)
ROA	0.0045	−0.0623	−0.0241
	(0.55)	(−0.97)	(−0.37)
Growth	−0.0011^***^	0.0015	0.0021
	(−2.63)	(0.58)	(0.80)
Top1	−0.0364^***^	−0.002	−0.0144
	(−4.27)	(−0.06)	(−0.39)
Cr_10	−0.0173^**^	−0.112^***^	−0.0720^**^
	(−1.99)	(−2.98)	(−2.06)
Soe	−0.0165^***^	−0.00200	−0.0036
	(−6.40)	(−0.18)	(−0.26)
Ins	−0.0063	0.0389	0.0061
	(−1.09)	(1.63)	(0.30)
Inde	0.0194	0.0646	0.0523
	(1.17)	(0.99)	(0.81)
Ind	Y	Y	Y
Year	Y	Y	Y
*N*	16,593	1,256	1,256
*R* ^2^	0.18	0.223	0.223

^***^, ^**^, ^*^ represent the significance level of 1, 5, 10%, respectively.

Column (2) shows that the EI_I regression coefficient is 0.0525, which is significant at the level of 1% (*β* = 0.0525, *p* < 0.01), indicating that in companies implementing executive equity incentive plans, increasing the incentive intensity will significantly improve the level of investor relations management, which supports Hypothesis 1. In an economic sense, for every unit increase in incentive intensity, the company’s investor relations management level increases by 5.25%. Based on the investor relationship management level, this result shows that under the equity incentive compensation system, executives are more inclined to obtain long-term benefits by optimizing the investor relationship management of listed companies, rather than short-term benefits such as on-the-job consumption and informed transactions. The direct benefits. At the same time, the increase in the intensity of equity incentives has led to further compression of management’s opportunism. The executives will promote the company to improve the management of investor relations based on the purpose of stock appreciation or improving asset liquidity.

Column (3) is the regression result of model (4). The regression coefficient of EI_M is 0.0236, which is significant at the 1% level (*β* = 0.0236, *p* < 0.01),. This shows that from the perspective of incentive models, the stock option model has a better incentive effect than the restricted stock model, and the regression results meet the expectations of Hypothesis 2. In addition, the variance expansion factor of each regression model is less than 5, indicating that the model does not have a serious collinearity problem.

### Robustness test

#### Propensity score matching

Since the implementation of EEIPs accounts for a relatively low proportion of the sample, in order to alleviate the possible endogenous problems in the empirical research and enhance the validity of the research conclusions, this paper uses the propensity score matching method (PSM) to test the robustness of the empirical results of executive equity incentive and Investor Relations Management. The regression model is as follows:

Model 2.


$${EI}_{i,t}=\alpha_{0}+\Sigma \alpha {\text{MatchingVariables}}_{i,t}+£\text{ Year}+£\text{ Industry}+\varepsilon$$

Among them, *α* is the regression coefficient, ***ε*** is the error term. EI is an dummy variable. If the listed company implements executive equity incentives, it is assigned as 1, otherwise it is 0. Because the characteristics of the company will have a significant impact on the implementation of equity incentives (Chourou et al., 2008). this paper selects the following factors as matching variables: Enterprise size (Size; Konari, 2005), asset liability ratio (Lev; Sun and Shen, 2015) and Nature of Property Rights (Soe; Wang et al., 2021).

This paper uses the nearest neighbor matching method according to the 1:1 matching method without replacement to find the control group companies for the companies that implement equity incentives. Table 6 reports the matching estimation results. ATT (Average Treatment Effect of Executive Equity Incentive Companies) is significant at the 1% level. At the same time, this paper conducted a balance test. The test results show that the standardized deviation of all covariates after matching is less than 10%, and the results of all t-tests do not reject the null hypothesis that the treatment group and the control group are not systematically different. Compare the results before matching. The standardization deviations of all variables are greatly reduced, indicating that all covariates have passed the balance test. This shows that after propensity score matching, the differences in characteristics between companies that implement executive equity incentives and those that do not implement executive equity incentives have been eliminated to a greater extent. The matching regression results are shown in column (1) in Table 7. The regression coefficient of EI is significantly positive at the 1% level, indicating that under the circumstance of alleviating endogenous problems, the implementation of executive equity incentives by listed companies will improve investor relations The assumption of management level still holds.

**Table 6:** Equity incentive (EI) and investor relations management (IRM): propensity score matching (PSM).

Variable	Sample	Treated	Controls	Difference	S.E.	*T*-stat.
IRII	Unmatched	0.3263	0.3009	0.0254	0.0037	6.76^***^
	ATT	0.3263	0.3047	0.0216	0.0054	3.97^***^

^***^, ^**^, ^*^represent the significance level of 1, 5, 10%, respectively.

**Table 7:** Robustness test of EEIPs and IRM.

	(1)	(2)	(3)	(4)	(5)	(6)	(7)
	IRII	First Stage	IV	IRII	IRII	IRII	IRII
Stock				0.135^***^			
				(3.50)			
EI	0.0167^***^		0.324***		0.0170^***^		
	(3.22)		(3.33)		(4.17)		
L3.EI		0.0799***					
		(4.19)					
EI_I						0.0648^***^	
						(2.89)	
EI_M							0.0259^**^
							(2.40)
Size	0.0498^***^	0.0068	0.0389***	0.0463^***^	0.0556^***^	0.0360^***^	0.0305^**^
	(7.27)	1.04	(11.64)	(4.60)	(20.70)	(3.06)	(2.56)
Equity	0.00566^***^	0. 0091***	0.00408***	0.00778^***^	0.00646^***^	0.00111	0.00737^**^
	(4.21)	(8.85)	(3.65)	(3.16)	(13.18)	(0.30)	(2.33)
Lev	−0.0515^***^	0. 0132	−0.0654***	−0.100^***^	−0.0525^***^	−0.0644^**^	−0.0645^**^
	(−2.87)	(0.83)	(−7.62)	(−3.67)	(−7.37)	(−1.98)	(−1.98)
ROA	−0.00551	0.1243***	0.0134	−0.0763	0.0119	0.00177	0.0459
	(−0.15)	(4.27)	(0.73)	(−1.18)	(1.20)	(0.02)	(0.63)
Growth	0.00295	0.0002	−0.0011**	0.00216	−0.000750	−0.00599	−0.00457
	(1.39)	(0.25)	(−2.29)	(0.83)	(−1.42)	(−0.96)	(−0.73)
Top1	−0.0353	−0.0077	−0.0172	−0.0197	−0.0385^***^	0.00670	−0.00411
	(−1.50)	(−0.27)	(−1.19)	(−0.58)	(−3.73)	(0.16)	(−0.10)
Cr_10	−0.0290	−0.0262	0.0834***	−0.168^***^	−0.0343^***^	−0.141^***^	−0.0894^**^
	(−1.23)	(−0.75)	(4.86)	(−3.76)	(−3.25)	(−3.11)	(−2.13)
Soe	−0.0305^***^	−0.0198**	−0.0103**	−0.00672	−0.0168^***^	−0.00193	−0.00381
	(−3.44)	(−2.69)	(−2.32)	(−0.50)	(−5.31)	(−0.12)	(−0.23)
Ins	−0.00522	0.0251	−0.0491***	0.0956^***^	−0.00315	0.0539^*^	0.0129
	(−0.36)	(1.02)	(−4.17)	(2.91)	(−0.46)	(1.89)	(0.53)
Inde	0.00343	0.1234**	−0.0160	0.0682	0.0130	0.0562	0.0409
	(0.08)	(2.15)	(−0.54)	(1.06)	(0.64)	(0.71)	(0.52)
Ind	Y	Y	Y	Y	Y	Y	Y
Year	Y	Y	Y	Y	Y	Y	Y
*N*	2,416	6,587	6,587	1,256	12,012	975	975
*R* ^2^	0.206	0.132	0.126	0.226	0.184	0.233	0.231

^***^, ^**^, ^*^represent the significance level of 1, 5, 10%, respectively.

#### Iv-2SLS

The previous article has confirmed that executive equity incentive plans will improve the level of investor relations management of enterprises, but it may also be that enterprises with better investor relations management are more inclined to implement EEIPs, that is, the previous conclusion may have an endogenous problem of reverse causation. In order to alleviate the endogenous problem, we constructs the instrumental variable based on the repeatability and intermittence of equity incentive in Chinese listed companies, and uses the three lagging periods (L3_EI) of EI as the instrumental variable, and uses 2SLS to test the robustness. In addition, in order to ensure the validity of the tool variable, firstly, we conducts an unrecognized test on it through Kleibergen-Paap rk LM statistic, and the result shows that the statistic value of p is 0.000, rejecting the original assumption that cannot be identified; secondly, we tested whether the tool variable is a “weak tool variable.” The result shows that the F statistic is significantly greater than 10, and the value of p is 0.000, indicating that the original hypothesis of “there is a weak tool variable” can be rejected. Columns (2) and (3) of Table 7 reports the IV-2SLS results. The results show that EEIPs will still improve the level of investor relationship management after controlling endogenous problems, which is consistent with the previous conclusion.

#### Replace main variable measurement

Based on the research of Lu et al. (2014) and Sheng et al. (2016), OLS regression was performed on the model again by using Stock as a substitute variable of executive equity incentive intensity. Column (4) of Table 7 reports the robustness test results, which are not substantially different from the results in this paper.

#### Reduce sample size

Since individual indicators of the investor relations interaction index were adjusted after 2015, only samples from 2016 to 2019 were selected for re-examination in order to avoid such influence. Columns (5)–(7) of Table 7 show the robustness test results of each hypothesis, which are basically consistent with the previous estimates.

## Further analysis: The impact path of EEIPs on IRM

As shown in Figure 1, IRII includes five secondary indicators: communication assurance (IRII_1), network communication (IRII_2), telephone communication (IRII_3), on-site communication (IRII_4) and communication feedback (IRII_5). Among them, communication guarantee refers to the organizational guarantee of communication with investors, including whether to set up investor relations department, professional background of the secretary and other indicators related to investor relations team construction; The middle three indicators all belong to the category of communication channels. Network communication and telephone communication, respectively, refer to online roadshows, teleconference and other activities carried out through the media of the Internet and telephone. On-site communication refers to the performance presentation, analyst meetings and investor research and reception activities carried out through face-to-face communication. Communication feedback includes email feedback. In order to further analyze the specific dimensions of EEIPs affecting the level of investor relationship management, this paper tests IRII’s five sub-indexes as explained variables, respectively.

Table 8 reports the test results of the impact of EEIPs on each sub-index of investor relationship management. In column (1), (2), and (5), communication assurance (IRII_1), network communication (IRII_2) and communication feedback (IRII_5) are explained variables, and EI coefficient is not significant. This shows that the implementation of executive equity incentive plan in listed companies has no significant impact on communication security index, network communication index and communication feedback index. Column (3) takes telephone communication (IRII_3) as the explained variable. The regression results show that the coefficient of EI is significantly positive at the level of 10%. This indicates that the telephone communication index of the enterprises implementing the executive equity incentive plan is higher than that of the enterprises not implementing executive equity incentive plans. Column (4) takes field communication (IRII_4) as the explained variable. The EI coefficients were significantly positive at 5% level. This indicates that the on-site communication index of the enterprises implementing EEIPs is higher than that of the enterprises not implementing EEIPs.

**Table 8:** The impact of EI on each sub-index of IRM.

	(1)	(2)	(3)	(4)	(5)
	IRII_1	IRII_2	IRII_3	IRII_4	IRII_5
EI	−0.000801	0.00592	0.0122^*^	0.0168^**^	0.00239
	(−0.12)	(1.17)	(1.85)	(2.24)	(0.64)
Size	0.0373^***^	0.0648^***^	0.00847^**^	0.0621^***^	0.0583^***^
	(9.12)	(20.09)	(2.02)	(12.99)	(24.69)
Equity	0.00603^***^	0.00923^***^	−0.0000717	0.0141^***^	0.00194^***^
	(8.15)	(15.85)	(−0.09)	(16.39)	(4.56)
Lev	−0.0269^**^	−0.0586^***^	−0.0121	−0.117^***^	−0.0452^***^
	(−2.53)	(−6.98)	(−1.10)	(−9.41)	(−7.36)
ROA	0.000718	−0.0274^**^	0.0204	0.0292^*^	−0.0177^**^
	(0.05)	(−2.47)	(1.41)	(1.78)	(−2.18)
Growth	0.000130	0.00325^***^	0.000162	−0.00134	0.000150
	(0.16)	(−5.18)	(0.20)	(−1.44)	(0.33)
Top1	−0.00929	−0.0374^***^	−0.0817^***^	−0.0858^***^	0.000923
	(−0.59)	(−3.01)	(−5.04)	(−4.66)	(0.10)
Cr_10	−0.00329	−0.0201	0.0223	0.0717^***^	−0.145^***^
	(−0.20)	(−1.58)	(1.34)	(3.80)	(−15.58)
Soe	−0.0153^***^	−0.0279^***^	−0.00784	−0.0275^***^	−0.00581^**^
	(−3.22)	(−7.42)	(−1.60)	(−4.94)	(−2.11)
Ins	0.0105	−0.0111	0.00585	−0.0378^***^	0.0116^*^
	(0.99)	(−1.33)	(0.54)	(−3.04)	(1.89)
Inde	−0.0462	0.0594^**^	0.0164	0.0585	0.0364^**^
	(−1.50)	(2.44)	(0.52)	(1.63)	(2.05)
Ind	Y	Y	Y	Y	Y
Year	Y	Y	Y	Y	Y
*N*	16,593	16,593	16,593	16,593	16,593
*R* ^2^	0.379	0.135	0.078	0.136	0.167

^***^, ^**^, ^*^represent the significance level of 1, 5, 10%, respectively.

Table 9 shows the test results of the influence of executive equity incentive intensity and equity incentive mode of listed companies on each sub-index of investor relationship management. Columns (1) and (6) take communication guarantee (IRII_1) as the explained variable, and the EI_I coefficient and EI_M coefficient are not significant, indicating that neither has a significant influence on the communication guarantee index. Column (2) and (7) take network communication (IRII_2) as the explained variable, and both EI_I coefficient and EI_M coefficient were significantly positive at the 5% level. The results show that the greater the executive equity incentive intensity, the higher the enterprise and investors’ network communication index, and the stock option equity incentive model is more conducive to improve the network communication index of listed companies. Columns (3) and (8) use telephone communication (IRII_3) as the explained variable. The regression results show that the EI_I coefficient is not significant, but the EI_M coefficient is significantly positive at the 1% level. It shows that the intensity of executive equity incentives does not have a significant impact on the telephone communication index, but the equity incentive model of stock options helps to improve the telephone communication index of listed companies. Column (4) and (9) take on-site communication (IRII_4) as the explanatory variable, and the EI_I and EI_M coefficients were significantly positive at 1 and 5% levels, respectively. The results show that the greater the intensity of executive equity incentives, the higher the corporate on-site communication index, and the equity incentive model of stock options can help improve the listed company on-site communication index. Columns (5) and (10) use communication feedback (IRII_5) as the explained variable, and neither EI_I coefficient nor EI_M coefficient is significant, indicating that neither of them has significant influence on the communication feedback index.

**Table 9:** The impact of EI_I and EI_M on each sub-index of IRM.

	(1)	(2)	(3)	(4)	(5)	(6)	(7)	(8)	(9)	(10)
	IRII_1	IRII_2	IRII_3	IRII_4	IRII_5	IRII_1	IRII_2	IRII_3	IRII_4	IRII_5
EI_I	0.00098	0.0592^**^	0.0180	0.117^***^	0.0284					
	(−0.03)	(2.38)	(0.52)	(3.02)	(1.45)					
EI_M						0.0174	0.0265^**^	0.0445^***^	0.0393^**^	0.00343
						(1.15)	(2.30)	(2.80)	(2.19)	(0.38)
Size	0.00645	0.0445^***^	0.0835^***^	0.0409^**^	0.0685^***^	0.00384	0.0382^***^	0.0761^***^	0.0305	0.0669^***^
	(0.37)	(3.37)	(4.57)	(1.99)	(6.60)	(0.22)	(2.88)	(4.15)	(1.48)	(6.40)
Equity	0.0123^**^	0.00262	−0.00400	0.00235	0.00133	0.0127^***^	0.00824^**^	−0.00137	0.0131^***^	0.00379
	(2.44)	(0.68)	(−0.75)	(0.39)	(0.44)	(2.97)	(2.54)	(−0.31)	(2.59)	(1.48)
Lev	0.00970	−0.104^***^	−0.184^***^	0.183^***^	0.0922^***^	0.0113	−0.102^***^	−0.181^***^	−0.181^***^	0.0925^***^
	(0.20)	(−2.88)	(−3.70)	(−3.27)	(−3.26)	(0.24)	(−2.85)	(−3.64)	(−3.24)	(−3.26)
ROA	0.00150	−0.0923	−0.0391	0.00610	−0.0773	0.0163	−0.0490	0.00569	0.0680	−0.0647
	(0.01)	(−1.09)	(−0.33)	(−0.05)	(−1.17)	(0.15)	(−0.58)	(0.05)	(0.52)	(−0.97)
Growth	0.000738	0.00128	0.00600	0.00734	0.000699	0.000924	0.00193	0.00660	0.00848	−0.00049
	(0.16)	(0.37)	(1.26)	(1.38)	(−0.26)	(0.20)	(0.56)	(1.39)	(1.59)	(−0.18)
Top1	0.186^***^	−0.139^***^	−0.0628	0.00776	0.0399	0.185^***^	−0.152^***^	−0.0695	−0.0316	0.0346
	(3.15)	(−3.10)	(−1.01)	(−0.11)	(1.13)	(3.14)	(−3.39)	(−1.13)	(−0.46)	(0.98)
Cr_10	−0.141^**^	−0.0752	0.0844	−0.184^**^	−0.226^***^	−0.142^**^	−0.0285	0.0987	−0.0917	−0.203^***^
	(−2.14)	(−1.51)	(1.22)	(−2.37)	(−5.74)	(−2.35)	(−0.62)	(1.56)	(−1.28)	(−5.62)
Soe	−0.0202	−0.0114	0.0313	−0.0253	0.0139	−0.0190	−0.0125	0.0332	−0.0284	0.0128
	(−0.86)	(−0.64)	(1.27)	(−0.92)	(0.99)	(−0.81)	(−0.70)	(1.35)	(−1.03)	(0.91)
Ins	0.0373	0.0506	−0.0491	0.0699	0.0270	0.0388	0.0136	−0.0585	−0.00365	0.00883
	(0.90)	(1.61)	(−1.13)	(1.43)	(1.09)	(1.09)	(0.50)	(−1.57)	(−0.09)	(0.41)
Inde	−0.0234	0.160^*^	−0.0232	0.316^**^	0.0103	−0.0299	0.147^*^	−0.0410	0.295^**^	0.00750
	(−0.21)	(1.89)	(−0.20)	(2.40)	(0.15)	(−0.27)	(1.73)	(−0.35)	(2.23)	(0.11)
Ind	Y	Y	Y	Y	Y	Y	Y	Y	Y	Y
Year	Y	Y	Y	Y	Y	Y	Y	Y	Y	Y
*N*	1,256	1,256	1,256	1,256	1,256	1,256	1,256	1,256	1,256	1,256
*R* ^2^	0.404	0.170	0.184	0.177	0.224	0.405	0.170	0.189	0.174	0.222

^***^, ^**^, ^*^represent the significance level of 1, 5, 10%, respectively.

To sum up, EEIPs mainly through strengthening the telephone communication and on-site communication improve the level of investor relations management, executive equity incentive intensity is mainly through enhancing network communication, field communication can improve the investor relations management level, executive equity incentive mode mainly through the network communication, telephone communication and field communication improved investor relations management level. However, the three indexes have no significant influence on communication security index and communication feedback index.

The above results indicate that the company has choice preference when choosing the investor communication path, which is mainly based on the consideration of cost and benefit. Investor-relationship management brings benefits to executives, but also brings high cost to enterprise operation (Hong and Huang, 2003; Song and Thakor, 2008). As a way of instant communication, network communication, telephone communication and on-site communication have the advantages of quick effect and low cost, and play the most direct role in the transmission of information. Companies are more inclined to adopt these communication strategies for information interaction with investors. In addition, compared with online communication and telephone communication, listed companies are more willing to improve investor relations through on-site communication after the implementation of executive equity incentive plans, which may be because on-site communication is more likely to improve investor confidence and help enterprises obtain market support (Bushee et al., 2011; Lin and Xie, 2016). Improving the communication guarantee mechanism is a long-term work, and the company needs to establish a professional investor relations team and make certain achievements in the field of investor relations. Therefore, equity incentive does not significantly improve the communication guarantee index in a short period of time. Communication feedback may involve email feedback, telephone frequency and other aspects, most of which need one-to-one targeted communication, which is complicated, requires more time and energy, relatively high cost and relatively slow effect. Therefore, the company is not willing to strengthen communication and feedback.

## Conclusion and policy implications

### Conclusion

We examine the impact of EEIPs on a firm’s investor relationship management. We argue that EEIPs directly increase firms’ investor relationship management because of the implementation of EEIPs mitigates agency conflicts between managers and shareholders and motivates managers to obtain excess returns from stocks, improve the liquidity of stocks held and the ability to realize assets, promoting the improvement of investor relations. Under the background of the comprehensive promotion of the reform of the registration system in China, the right of the value judgment of listed companies is being more transferred to the investors themselves, and the closeness of the relationship between enterprises and investors is considered to be a key factor to improve the reputation and value of enterprises. At the same time, the number of equity incentive plans issued by listed companies in China also reached a peak. We wonder whether there is a connection between the both. Is the substantial increase in the number of equity incentive plans a response of the market to the reform of the registration system? In response to this phenomenon, we hypothesize that, firms with EEIPs respond by engaging relatively more in investor relationship management activities. Based on the panel data of Chinese listed companies from 2014 to 2019, our findings support this hypothesis. Specifically, we document that firms with EEIPs have greater investor relations management compared to those without EEIPs and high intensity EEIPs are accompanied by more positive investor relations interaction. As for the form of EEIPs, we find that stock options plays a major role in IRM, while restricted stock have no impact. For the analysis purpose, this paper employs fixed-effect (FE) model was used in this paper. In addition, For more robust results, this paper employs the propensity score matching (PSM), instrumental variable method, and core indicator substitution method to test the robustness of the conclusions. Further research shows that EEIPs mainly through telephone communication, network communication and on-site communication to achieve the impact of listed companies investor relationship management, among which enterprises are more inclined to adopt on-site communication to interact with investors. Considering the time span and complexity of some investor relations work, executive equity incentive has no significant influence on the communication guarantee index and communication feedback index.

This paper provides further empirical evidence for the existing research on how to stimulate enterprises’ investor relationship management, explores the internal effective ways to stimulate enterprises’ investor relationship management. At the same time, this paper also enriches the external channels that equity incentive affects enterprise value. In addition to having an impact on the internal governance of the enterprise, equity incentive will also attract more attention from external entities. External entities will play an external governance role, which will ultimately have an impact on the enterprise value. As an important influencing factor of stock pricing of listed companies, investor relations management research needs to be further expanded. On the basis of previous discussions on “equity incentive to attract analysts to track,” “equity incentive to increase the frequency of management performance forecast” and “equity incentive to increase the number and scale of investor research,” this paper puts forward the idea of “equity incentive to improve investor relationship management.” Through the investor relationship management index (IRII), we can more comprehensively judge the impact of equity incentive on investor relations. Furthermore, this paper studies the impact of equity incentive on different dimensions of investor relationship management, enriching and refining relevant research, and supplemented the application scenarios of optimal contract theory in compensation incentives and investor relations.

### Policy implications

Based on our research, we propose the following implications, and policymakers, firm owners, firm management, and investors in Chinese will benefit from this research.

First, for listed companies, EEIPs should be incorporated into the executive compensation system to enhance the two-way communication between enterprises, shareholders and investors. The combination of executive equity incentive plans and investor relationship management is conducive to accumulating reputation of capital market, improving investor confidence and optimizing market value management.

Second, under the background of registration system reform, it is the trend to actively promote the improvement of investor relations management. For the regulatory authorities, it is necessary to improve the equity incentive mechanism, encourage listed companies to promote EEIPs, guide enterprises to establish two-way communication channels and environment for investors, improve the quality of information disclosure, and promote the stable and healthy development of the capital market.

Third, for investors, EEIPs of listed companies can be taken as an important reference index for investment decisions. Specifically, equity incentive plans for top executives of listed companies will lead to warmer investor relations, a sign that corporate policymakers are willing to maintain good relations with investors and be monitored. The transparency of information and the potential supervision mechanism mean that the probability of management’s opportunistic behavior is relatively small, and the company is more worthy of investment by investors.

## Limitations and future directions

This paper used data for a limited period from 2014 to 2019 due to data availability issues. Therefore, this period could be extended with the availability of the data. For future research, the other major elements of corporate governance such as CEO heterogeneity and managerial ownership, etc. can also be taken as moderating factors to investigate the connection between executive equity incentive plans and investor relationship management.

## Data availability statement

Publicly available datasets were analyzed in this study. This data can be found at: https://www.gtarsc.com/ (China Stock Market & Accounting Research Database, CSMAR).

## Author contributions

XS designed the paper, collected the data, summarized the literature review, developed the hypotheses, conducted the empirical analysis and finalized the manuscript. ZL contributed to the hypothesis development and performed the empirical analysis. YW provided valuable suggestions and comments regarding the manuscript. All authors contributed to the article and approved the submitted version.

## Funding

This study was supported by National Natural Science Foundation of China (72172063), S&T Program of Hebei (22557607D), and Humanities and Social Science Research Project of Hebei Education Department (SD2022054).

## Conflict of interest

The authors declare that the research was conducted in the absence of any commercial or financial relationships that could be construed as a potential conflict of interest.

## Publisher’s note

All claims expressed in this article are solely those of the authors and do not necessarily represent those of their affiliated organizations, or those of the publisher, the editors and the reviewers. Any product that may be evaluated in this article, or claim that may be made by its manufacturer, is not guaranteed or endorsed by the publisher.
